# Use of *Propionibacterium freudenreichii* T82 Strain for Effective Biosynthesis of Propionic Acid and Trehalose in a Medium with Apple Pomace Extract and Potato Wastewater

**DOI:** 10.3390/molecules26133965

**Published:** 2021-06-29

**Authors:** Kamil Piwowarek, Edyta Lipińska, Elżbieta Hać-Szymańczuk, Anna Maria Kot, Marek Kieliszek, Sylwia Bonin

**Affiliations:** Department of Food Biotechnology and Microbiology, Institute of Food Sciences, Warsaw University of Life Sciences—SGGW, Nowoursynowska 159 C, 02-776 Warsaw, Poland; edyta_lipinska@sggw.edu.pl (E.L.); elzbieta_hac_szymanczuk@sggw.edu.pl (E.H.-S.); anna_kot@sggw.edu.pl (A.M.K.); marek_kieliszek@sggw.edu.pl (M.K.); sylwia_bon@gmail.com (S.B.)

**Keywords:** waste, apple pomace, glycerine, potato wastewater, *Propionibacterium*, propionic acid, trehalose

## Abstract

Propionic acid bacteria are the source of many metabolites, e.g., propionic acid and trehalose. Compared to microbiological synthesis, the production of these metabolites by petrochemical means or enzymatic conversion is more profitable. The components of microbiological media account for a large part of the costs associated with propionic fermentation, due to the high nutritional requirements of *Propionibacterium*. This problem can be overcome by formulating a medium based on the by-products of technological processes, which can act as nutritional sources and at the same time replace expensive laboratory preparations (e.g., peptone and yeast extract). The metabolic activity of *P. freudenreichii* was investigated in two different breeding environments: in a medium containing peptone, yeast extract, and biotin, and in a waste-based medium consisting of only apple pomace and potato wastewater. The highest production of propionic acid amounting to 14.54 g/L was obtained in the medium containing apple pomace and pure laboratory supplements with a yield of 0.44 g/g. Importantly, the acid production parameters in the waste medium reached almost the same level (12.71 g/L, 0.42 g/g) as the medium containing pure supplements. Acetic acid synthesis was more efficient in the waste medium; it was also characterized by a higher level of accumulated trehalose (59.8 mg/g d.s.). Thus, the obtained results show that *P. freudenreichii* bacteria exhibited relatively high metabolic activity in an environment with apple pomace used as a carbon source and potato wastewater used as a nitrogen source. This method of propioniate production could be cheaper and more sustainable than the chemical manner.

## 1. Introduction

Propionic acid bacteria (PAB) are Gram-positive, nonsporulating rods. They are classified as anaerobic or relatively anaerobic. The optimal growth conditions for *Propionibacterium* are pH in the range of 6.0–7.0 and temperature in the range of 30–37 °C. *Propionibacterium freudenreichii* have been used in cheese production, forage silage, and animal nutrition as probiotics. PAB are the source of many valuable metabolites, including propionic acid, B vitamins (e.g., B12), bacteriocins, trehalose, and glycogen [1]. 

The annual production of propionic acid is over 400,000 tonnes, and the global tycoon is the German company BASF [2]. For industrial purposes, propionate is synthesized by petrochemical means—Reppe and Larson processes. The Reppe process involves the conversion of ethylene, carbon monoxide, and steam into propionic acid, while in the Larson process, propionic acid is produced from ethanol and carbon monoxide in the presence of a catalyst (boron trifluoride) [2,3]. Propionic acid is used primarily in the food and feed industry as a preservative for inhibiting the growth of yeasts and molds. Moreover, propionic acid and its derivatives are used as substrates in the production of cellulose fibers, herbicides, perfumes, and pharmaceuticals [4].

Trehalose occurs in plants, invertebrates, and microorganisms including the cells of PAB. On an industrial scale, trehalose is produced by the transformation of maltodextrins or starch (from potatoes, corn, and tapioca), which is catalyzed mainly by enzymes isolated from *Arthobacter ramosus* Q36 bacteria-malto-oligosyltrehalose synthase and malto-oligosyltrehalose trehalohydrolase. The use of enzymatic transformation led to a significant reduction in the price of trehalose (from $100 to $5–6 per kilogram) and an increase in its availability on the market. At present, the annual production of trehalose is approximately 30,000–50,000 tonnes [5]. Due to its unique properties, trehalose has a wide range of applications. Trehalose is half as sweet as sucrose and reduces insulin secretion, which makes it ideal for producing food products for diabetics [4]. Trehalose is also a component of eye drops (to treat dry eyes), solutions used to hold tissues, organs, enzymes, and vaccines. Furthermore, it is used in creams and cosmetic lotions to retain moisture or strengthen their stability during storage [6]. Moreover, attempts have been made to use trehalose in the treatment of osteoporosis and neurodegenerative conditions such as Alzheimer’s, Parkinson’s, and Huntington’s disease [7,8,9,10].

Growing social awareness and care for the environment have together contributed to an increased demand for ecological food and cosmetic products. An interesting alternative, which enables economical as well as sustainable production of industrially valuable substances, seems to be the biotechnological utilization of industrial waste with the use of microorganisms such as PAB [11,12,13,14,15,16]. They have GRAS and QPS statuses, which indicates that both living cells and metabolites of these microorganisms can be used in the production of food, cosmetics, and drugs. 

The food industry generates a significant amount of sewage and solid waste and thus poses a serious threat to the natural environment. The management of this waste is troublesome and costly. For this reason, there is a search for effective methods of their disposal that are both environmentally safe and commercially remunerative. The use of PAB for waste management can be beneficial, as it enables obtaining the metabolites of these microorganisms. This may allow eliminating the petrochemical route of propionic acid synthesis as well as reducing the production costs of individual compounds and finally target products (waste is widely available and cheap, is a reservoir of biologically active compounds such as sugars, nitrogen sources, vitamins, and minerals, and can thus reduce media costs). In addition, consumers can be provided with products of microbiological origin without chemical synthesis. However, the most important thing is that it can allow for efficient waste management, improving the quality of the natural environment. This is particularly important considering the fact that chemical processes require non-renewable petrochemical raw materials and also pollute the environment.

Apple processing leads to the generation of solid waste and sewage. The amount of apple pomace depends on the variety of apples and pressing technology used, making up 10–30% of the weight of the fruits [17]. It mainly contains water, which constitutes about 70% of the mass of this waste. The dry matter of pomace comprises saccharides, proteins, minerals, pectin, fiber, lipids, organic acids, vitamins, and colored and aromatic substances [18]. Apple pomace is used as an animal feed component and can also be used in biogas plants. The conservation of pomace by freezing or drying involves equipment (purchase, service) and energy costs. Apple pomace is an unstable material and susceptible to microbiological contamination in the fresh state, mainly due to its high content of water and biologically active compounds [19,20,21]. The uncontrolled growth of microorganisms in apple pomace may result in the loss of its valuable technological properties, including the content of nutrients. Therefore, an alternative solution is to subject fresh pomace to biotechnological utilization immediately after the pressing process, e.g., disposal with PAB.

Potato wastewater is obtained during the production of potato starch. In Europe, about 1.7 million tonnes of potato starch is produced annually. It is estimated that the processing of 1000 tonnes of potatoes leads to the production of about 600 tonnes of potato wastewater. Potato wastewater contains about 2.9–4.3% of dry substance, of which protein constitutes 0.93–1.57%, sugars 0.5–0.8%, fat 0.2%, and minerals 1%. It is also a rich source of protein and vitamins, mainly from the B group. Potato wastewater has no specific application—it is most often used to fertilize fields as a source of nitrogen absorbed by plants. However, potato wastewater has a high pollution load, and so using this waste as a fertilizer may lead to water eutrophication and deterioration of soil fertility [22,23]. Therefore, there is still a need to search for new, effective ways of managing this waste.

Most scientific reports describe that PAB can be cultivated in an environment containing waste materials, which often requires supplementation with expensive sources of nitrogen or other elements in the form of pure laboratory preparations. Earlier studies [11,12] used apple pomace as the carbon source but peptone and yeast extract as the nitrogen source. Taking into account the efficiency of the process and the costs incurred, it was shown that the production of propionate under these conditions is unprofitable (price of 1 kg of propionate = $3) [12], as the costs of substrate components (peptone, yeast extract, biotin, and mineral salts) are very high. This led to the need to investigate whether it is possible to replace expensive supplements with a much cheaper raw material that is quite dangerous for the environment, such as potato wastewater, which is a rich source of nitrogen, minerals, and vitamins. Therefore, the present study aimed to analyze the ability of a selected strain of PAB to grow in a medium containing agricultural and food industry waste both as carbon and nitrogen sources (apple pomace and potato wastewater), as well as the synthesis of propionic acid and accumulation of trehalose.

In this study, three variants of flask culture were carried out, and the following media were used: (1) apple pomace only; (2) apple pomace and pure laboratory preparations (yeast extract, peptone, biotin); (3) apple pomace and potato wastewater. Moreover, two different concentrations of carbon sources were used (2% and 4%) for each variant. The concentrations used resulted from the sugar content in the tested waste materials. 

## 2. Results and Discussion

### 2.1. Composition of Apple Pomace and Potato Wastewater

Potato wastewater was characterized by a higher pH and nitrogen content compared to apple pomace (Table 1). A total of 100 mL of potato wastewater had 0.214 g of nitrogen, while 100 mL of pomace extract had only 0.022 g. The total sugar content in the apple pomace extract was 4.54 g/100 mL. The share of fructose was highest in the total sugar content (53%), followed by glucose (42%) and sucrose (5%). On the other hand, only 0.51 g of sugars was found in 100 mL of potato wastewater. Similar to this study, Magyar et al. [20] observed in their study that most of the sugars in the used pomace were glucose and fructose, with sucrose accounting for 1%. In turn, in the pomace used by Skinner et al. [21], sugars accounted for up to 57% of the composition, most of which was composed of fructose (44%) and glucose (approximately 18%). A common feature of all the apple pomace samples studied was the negligible amount of protein. In the potato wastewater used in the studies by Kot et al. [24,25], the concentration of nitrogen was 0.206 and 0.232 g/100 mL, respectively, and that of direct reducing sugars was 1.38 and 0.73 g/100 mL, respectively. It should be remembered that the chemical composition of waste depends on, among other things, the variety of the raw material, climatic and atmospheric conditions during cultivation, harvest time, and method used for raw material storage or processing [26]. 

The molar ratio of carbon to nitrogen was 0.5:1 in potato wastewater and 39.2:1 in apple pomace. This result shows that potato wastewater can be used as a source of nitrogen and thus as a substitute for peptone or yeast extract, which is quite expensive. In turn, due to the sugar content, apple pomace can act as a source of carbon for microorganisms. However, the individual use of potato wastewater or apple pomace will not provide beneficial results because of the low content of separate substrates in these wastes—nitrogen in the case of pomace and sugar in the case of potato wastewater. It would result in a very high or very low molar ratio of carbon to nitrogen in the environment, which in turn would limit the metabolic activity of microorganisms [11]. A previous study has already described that the type and concentration of nitrogen and vitamin sources have an impact on the metabolic activity of PAB [27,28]. Fröhlich-Wyder et al. [29] also showed that the deficiency of aspartic acid in the medium inhibited the growth of *Propionibacterium*. In addition, some amino acids (mainly arginine and aspartic acid) act as a buffer, stabilizing the metabolism of PAB and enhancing the fermentation kinetics by reducing the inhibitory effect of acids on these bacteria [30]. 

### 2.2. Consumption of Carbon Sources and Growth of P. freudenreichii T82 Strain

**Figure 1:**
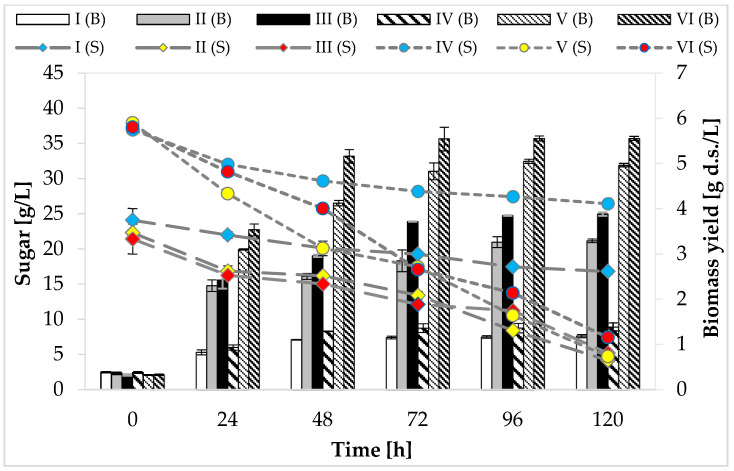
Consumption of carbon sources and growth of *P. freudenreichii* T82 strain. Reducing sugars (glucose and fructose) were included in the graph; sucrose consumption was not taken into account, because the tested strain does not assimilate and does not ferment this disaccharide/the sucrose content in individual media: (g/100 mL): I—0.08 ± 0.02; II—0.09 ± 0.01; III—0.07 ± 0.00; IV—0.15 ± 0.02; V—0.16 ± 0.03; VI—0.15 ± 0.01.

At the beginning of the cultivation, media I, II, and III contained approximately 20 g/L of reducing sugars (glucose and fructose in total). The maximum utilization of carbon sources by the tested strain, regardless of the medium variant, was noticed at 120 h of cultivation. It was found that none of the analyzed variants consumed the total amount of sugars available in the substrate (Figure 1). The greatest utilization of glucose and fructose (81%, 18.08 g/L) was observed in the medium with peptone, yeast extract, and other supplements (medium II). Media IV, V, and VI were characterized by a higher concentration of carbon sources—glucose and fructose were present in the range of 3.7–3.9%. The maximum use of carbon sources (reducing sugars) was recorded on the last day of fermentation in medium V (88%, 33.39 g/L). 

The highest biomass yield (5.55 g d.s./L) of the studied strain was found at 72 h of cultivation in media VI containing apple pomace and potato wastewater (Figure 1). The weakest growth of *P. freudenreichii* T82 strain, regardless of the concentration of carbon sources, was found in the media consisting only of apple pomace extract (medium I—1.16 g d.s./L, medium IV—1.36 g d.s./L). It was due to the trace amount of nitrogen and the high carbon-to-nitrogen molar ratio (36.6:1 and 34.9:1, respectively) in these media. 

Based on the obtained results, it can be concluded that *P. freudenreichii* T82 strain was able to grow in an environment consisting of apple pomace extract and potato wastewater. Moreover, the largest increases in the biomass of this tested strain were recorded in the breeding environment containing potato wastewater, which shows that the nitrogen sources in the studied waste were well absorbed by PAB (amino acids, amines, peptides). The better bacterial growth observed in the medium containing potato wastewater may have resulted, for example, from the greater availability of more suitable nitrogen sources compared to yeast extract or peptone (e.g., arginine or aspartic acid) [29]. This might be also related to the fact that the molar ratio of carbon to nitrogen in the medium with potato wastewater was lower compared to the medium containing yeast extract, peptone, and other supplements, indicating a higher nitrogen content, which directed bacterial metabolism to biomass formation. The production of carboxylic acids (e.g., propionic acid) takes place when bacteria are provided with favorable growth conditions—an excess amount of carbon and a limited amount of nitrogen. On the other hand, when the medium contains too much nitrogen, most of the carbon is used by bacteria for cell growth instead of acid biosynthesis [31].

### 2.3. Propionic Acid Production

**Figure 2:**
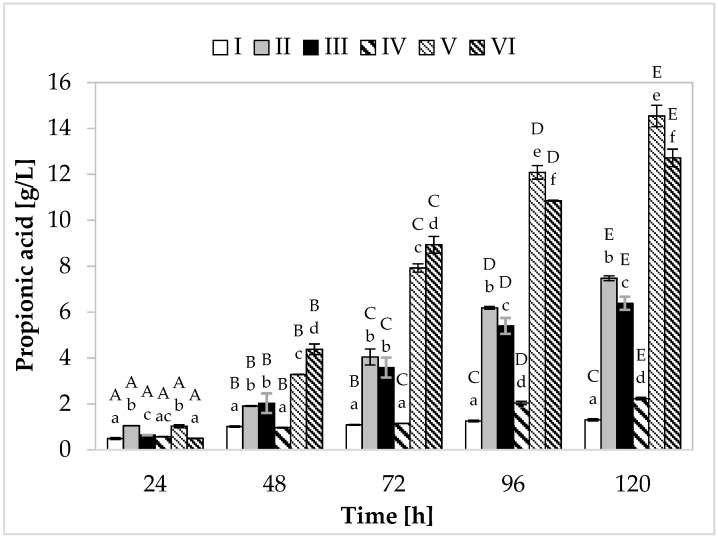
Propionic acid production. A, B, C, D, and E—homogeneous groups of the influence of the cultivation time on the production of propionic acid—the Tukey’s test (a one-way analysis of variance was performed); a, b, c, d, e, and f—homogeneous groups of the influence of the medium on the production of propionic acid—the Tukey’s test (a one-way analysis of variance was performed).

**Figure 3:**
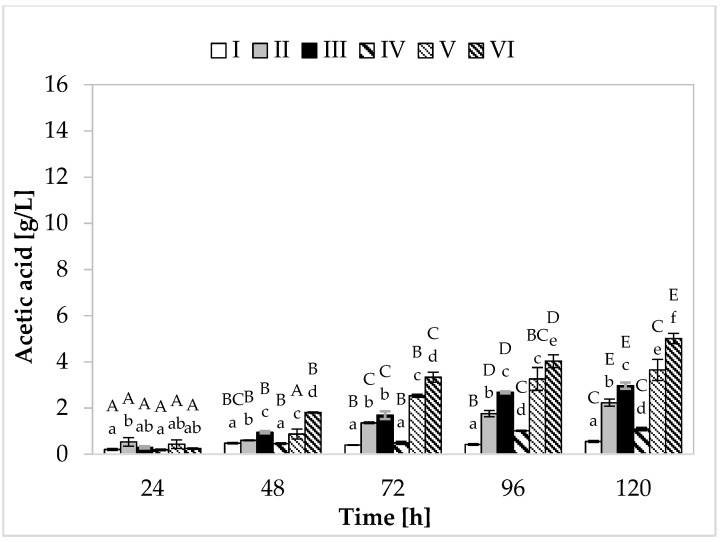
Acetic acid production. A, B, C, D, and E—homogeneous groups of the influence of the cultivation time on the production of acetic acid—the Tukey’s test (a one-way analysis of variance was performed); a, b, c, d, e, and f—homogeneous groups of the influence of the medium on the production of acetic acid—the Tukey’s test (a one-way analysis of variance was performed).

Regarding propionic acid production (Figure 2), in the medium containing yeast extract, peptone, and other supplements, the cultivation variant with lower sugar content, the maximum synthesis (7.48 g/L) was found at 120 h of fermentation. In the medium with apple pomace and potato wastewater, the bacteria produced on average 6.39 g/L of propionate (Figure 3). In media with higher sugar content, an increased yield of both acids was observed. The greatest amount of propionic acid (14.54 g/L) was formed in medium V containing yeast extract and peptone, while in the environment with only waste materials (apple pomace and potato wastewater), the level of this metabolite reached 12.72 g/L. The maximum acetic acid production (5.01 g/L) was found in the medium with potato wastewater (with approximately 4% content of carbon sources) at 120 h of fermentation.

The highest efficiency of propionic acid production was observed in medium V (variant with higher sugar content) (Table 2). In this medium, bacteria produced 0.44 g of propionic acid from 1 g of carbon source, with relatively low acetic acid production efficiency (0.11 g/g). Among the variants with potato wastewater, the best results were also achieved in the medium with 4% sugar content, with 0.42 g of propionic acid produced from 1 g of carbon source. Importantly, in the media containing this waste, higher efficiency of acetic acid synthesis was noted (medium III—0.18 g/g, medium VI—0.16 g/g). In the case of *Propionibacterium*, propionic acid production is closely related to cell growth. Both propionate production and cell growth processes are associated with pathways that involve phosphorylation at the substrate level and balance intracellular redox (glycolysis, pentose phosphate pathway, Wood–Werkman pathway). During the formation of cellular biomass and synthesis of propionic acid (pyruvate being the substrate in both cases), NADH is oxidized to NAD+ (during biomass formation, 5.75 moles of NAD+ are formed, while during acid production, two moles are formed). The media with potato wastewater (regardless of the sugar concentration) showed a higher yield of biomass than media II and V and efficient production of propionic acid, which probably resulted in more efficient synthesis of acetic acid. To maintain the intracellular redox balance, PAB produce a compensating metabolite in the form of acetic acid, during which NAD+ is reduced to NADH [32,33]. 

The most favorable weight ratio of propionic acid to acetic acid was noted in the media containing yeast extract and peptone (3.5:1 and 3.9:1) (Table 2). It should be remembered that the extraction of propionic acid from the culture fluid is inhibited by acetic acid. Thus, a low concentration of acetic acid in the medium increases the efficiency of propionic acid extraction. Although acetic acid reduces the yield of propionic acid, its production cannot be avoided in the case of some substrates. Since glycolysis cannot provide enough NADH for the synthesis of propionic acid (with glucose as the carbon source), acetic acid production provides an additional reduction reaction to maintain the intracellular redox balance. The production of propionic acid from glucose, which has a lower degree of reduction than propionic acid, requires the co-production of a more oxidized metabolite (e.g., acetic acid). Therefore, to improve the efficiency of propionate production and its extraction, it is necessary to limit the synthesis of acetic acid. In this context, media with yeast extract and peptone are more preferable (high P/A ratio and low acetic acid production yield). 

The yield of propionic acid production from apple pomace and potato wastewater was found to be relatively high. However, the fermentation of these waste materials could be further improved to reduce the synthesis of acetic acid, which would result in higher propionate production efficiency and improve the P/A weight ratio in the postculture fluid. This can be achieved by using waste glycerin as an additional carbon source. Glycerol is characterized by the same degree of reduction as propionic acid, and so its conversion to pyruvate can provide sufficient NADH for the biosynthesis of propionic acid without the need to co-produce acetic acid as a compensating metabolite. A much more favorable balance of propionic acid production using nutrients supplemented with yeast extract and peptone may result from additional enrichment with biotin [11]. Biotin is a cofactor that plays an important role in the synthesis of propionic acid. It takes part in the transcarboxylation reaction catalyzed by the enzyme methylmalonyl-CoA carboxyltransferase, which transfers the carboxyl group from methylmalonyl-CoA to pyruvate-forming oxalacetic acid and propionyl-CoA. It was assumed that the optimal concentration of this cofactor in the medium increased the affinity of the Wood–Werkman pathway for pyruvate, resulting in a higher yield of propionate and a more favorable weight ratio of metabolites. However, in this study, the media containing potato wastewater were not supplemented with biotin because the objective was to analyze how PAB would cope in an environment consisting entirely of waste. 

Various sources of nitrogen were used for the cultivation of PAB, both in the form of laboratory preparations (yeast extract, peptone, (NH_4_)_2_SO_4_) and industrial waste (corn pulp, sugar cane pomace, soy molasses, liquid acid protein residues from soybeans, by-product of the wet corn milling process, sweet sorghum bagasse, and corn steep liquor). In the case of waste, the by-product often served simultaneously as a source of carbon, nitrogen, and other components necessary for the efficient metabolism of microorganisms [13,14,27,34,35,36,37,38]. This study shows that apple pomace, due to its low protein content, is a poor nitrogen source and therefore cannot be used as the only ingredient. The production efficiency of propionic acid observed in the medium solely consisting of pomace was only 0.21 g/g. This indicates that this waste should be additionally enriched. Supplementation of pomace with yeast extract and peptone improves the efficiency of propionic acid production (0.41–0.44 g/g), but it is unprofitable. According to Piwowrek et al. [12], the market price of 1 kg of propionic acid would then be approximately $3 (mainly due to the costs associated with supplementation), while the current market price of 1 kg of chemically produced propionate is $1–2. Therefore, for the competitive biotechnological synthesis of propionic acid using *Propionibacterium* bacteria and apple pomace, a cheaper substitute of nitrogen source should be used. This study shows that potato wastewater (0.39–0.42 g/g) may be a promising nitrogen source. The overall goal of the study was to use a waste material that is cheap or completely worthless (due to the lack of management) for the ecological production of compounds of industrial importance, which would then have a lower market price due to reduced production costs (by using waste as substrates). In addition, the processing of bio-based waste into industrially valuable substances is associated with a number of other advantages, such as a less negative impact on the environment, waste disposal, and reduced energy as well as water requirements.

The use of potato wastewater as a source of nitrogen, vitamins, and minerals in the apple pomace medium seems quite promising compared to other waste media used so far. Lower or similar yields of propionic acid by PAB were recorded with the use of Jerusalem artichoke hydrolyzate (efficiency with free cells—0.38 g/g, efficiency with immobilized cells—0.43 g/g) [39], soybean molasses (or its hydrolyzate) with corn steep liquor (0.39, 0.42, and 0.46 g/g—depending on the cultivation variant) [37], and maize straw hydrolyzate (0.44 g/g) [35]. On the other hand, scientific reports describe a significantly higher yield of propionate using PAB and waste. Fermentation of sweet bagasse hydrolyzate from sorghum resulted in a propionic acid yield of 0.51 g/g [13]. It should be noted, however, that such efficiency was obtained by supplementing the waste with yeast extract, soybean tryptic broth, K_2_HPO_4_, and MnSO_4_, which ultimately results in a significant increase in production costs. The maximum yield of propionate from pomace and potato wastewater (without additional supplementation) was 0.42 g from 1 g of carbon sources. Such a yield was ensured by the culture of free cells in flasks—it can therefore be assumed that, as in the above-mentioned works, the use of immobilized cells of *P. freudenreichii* T82 and cultivation in a bioreactor environment would intensify the synthesis of propionic acid. An advantage of using apple pomace and potato wastewater is that these waste materials do not require enzymatic hydrolysis, and the carbon contained in them is directly digestible by PAB. This saves the costs associated with the enzymatic hydrolysis process (time, water, energy, equipment, and purchase of enzymes).

### 2.4. Treahlose Accumulation

There are few known pathways for the metabolism of trehalose—OtsAB, TreS, TreYZ, TreP, and TreT—of which the first two are used by *Propionibacterium*. According to a report from 2007, *P. freudenreichii* species synthesize this compound via the OtsAB pathway, while the TreS pathway is responsible for its catabolism [40,41]. The OtsAB pathway involves two enzymatic steps that are catalyzed by trehalose-6-phosphate synthase (OtsA) and trehalose phosphatase (OtsB), respectively [42]. PAB accumulate trehalose in response to osmotic, oxidative [43], acidic, or thermal stress [40,42]. Under stressful conditions, trehalose maintains cell turgor (in the case of increased osmotic pressure) or stabilizes cell membranes (proteins and body membranes) when cells have to withstand a strong pH gradient in an acidic environment. 

**Figure 4:**
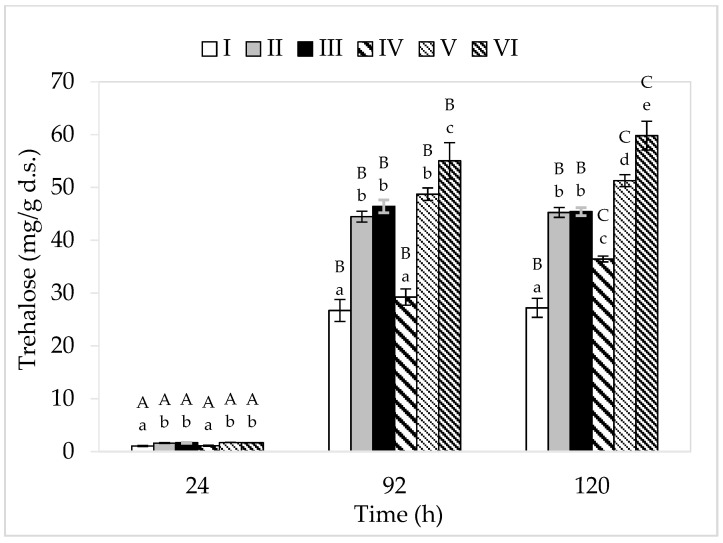
Accumulation of trehalose. A, B, and C—homogeneous groups of the influence of the cultivation time on the accumulation of trehalose—the Tukey’s test (a one-way analysis of variance was performed); a, b, c, d, and e—homogeneous groups of the influence of the medium on the accumulation of trehalose—the Tukey’s test (a one-way analysis of variance was performed).

Among the analyzed cultivation variants, the lowest concentration of this disaccharide was found in the cell biomass of bacteria grown in media I and IV (consisting only of apple pomace extract) (Figure 4). This may be related to the limited bacterial metabolism in these breeding conditions due to low nitrogen content, and thus a slight increase in the tested strain. The greatest amount of trehalose (59.80 mg/g d.s.) was accumulated by *P. freudenreichii* in medium VI (apple pomace and potato wastewater). The highest yield of cellular biomass (5.55 g d.s./L) was also recorded in this medium variant. Ruhal and Choudhury [42] showed that the higher the cellular biomass yield, the larger the amount of intracellularly accumulated trehalose. In this study, in the media with lower sugar content, trehalose accumulation lasted for up to 96 h of culture, while in media with a higher concentration of carbon sources, it lasted for up to 120 h. In the cultivation variants IV–VI (higher sugar content), a higher trehalose content was found in the bacterial cell biomass compared to the corresponding substrates I–III (lower sugar content). This is probably due to the presence of a higher amount of sugar in these breeding media and thus the higher osmotic pressure. Trehalose is accumulated by bacteria in logarithmic and stationary growth phases, and apart from its protective function, this disaccharide also acts as a reserve material. The accumulation of trehalose in the cell biomass of PAB occurs due to the action of stressors on microbial cells. An important factor is also the availability of carbon sources in the medium, which can be transformed by microorganisms into trehalose (cells must be well-nourished). In this study, regardless of the content of carbon sources, none of the analyzed cultivation variants showed loss of previously accumulated trehalose, despite the fact that it serves as a reserve material. This is due to the fact that in none of the media were the available carbon sources (glucose and fructose) completely utilized. Throughout the period of cultivation, the bacteria had access to sugars from waste, so they did not have to use the accumulated trehalose. Cardoso et al. [44] showed that only after depletion of glucose or lactose did PAB start using the trehalose sugar accumulated during the fermentation process.

Compared to the literature, the content of trehalose determined in the cell biomass of *P. freudenreichii* T82 in the present study is relatively low. However, it should be remembered that optimal culture parameters were used in the study for the synthesis of propionic acid by *P. freudenreichii* T82 (pH: 6.5–7.0, culture temperature: 37 °C) [11]. The accumulation of trehalose in the bacterial cell biomass in each of the media variants indicated that the microorganisms, after all, were exposed to some stress factor. The research conducted by Cardoso et al. [44] showed that lowering the pH from 7.0 to 4.7 resulted in an almost twofold intensification of trehalose accumulation (increase in the content from approximately 200 mg/g d.s. to approximately 400 mg/g d.s.). Admittedly, in this study, the substrates were neutralized every 24 h, but between successive neutralization, the pH values decreased, even up to approximately 5.0—which could be a stress factor for PAB. It can be assumed that the accumulation of organic acids in the media between successive neutralization stimulated *P. freudenreichii* T82 to accumulate trehalose. A common feature of the tested media was that they all contained apple pomace extract, which could have been a source of stress factors, for example, heavy metals, residues of plant protection products, or compounds that increased the osmotic pressure of the breeding environment (e.g., pectin). The highest amount of trehalose was found in the bacterial biomass obtained in the medium containing apple pomace and potato wastewater (medium VI). Therefore, it can be concluded that potato wastewater increased the content of trehalose in the biomass of the tested strain. This could be related, for example, to the toxic glycoalkaloid solanine present in this waste. Moreover, the waste could contain substances capable of increasing the osmotic pressure of the breeding environment. Pawlicka-Kaczorowska and Czaczyk [45] showed that with the increase in the osmolarity of the medium, the accumulation of trehalose in the cell biomass of *P. freudenreichii* 1 strain also increased (2% glycerol—26.55 mg/g d.s., 5% glycerol—51.23 mg/g d.s., and 8% glycerol—55.81 mg/g d.s.). Metabolism, and thus the accumulation of trehalose, is an individual feature of each strain or species, and thus the level of synthesis of this compound differs between microorganisms. In their study, Pawlicka-Kaczorowska and Czaczyk [45] cultured four different strains of PAB (*P. freudenreichii* 1, *Acidipropionibacterium acidipropionici* 9, *Acidipropionibacterium jenesii* 9, and *P. freudenreichii* DSM 4902) under the same conditions (5% glycerol, 30 °C, 3 d of cultivation, initial pH = 7.0/no neutralization) and obtained different yields of trehalose (50.44, 32.57, 17.36, and 16.86 mg/g d.s., respectively).

## 3. Materials and Methods

### 3.1. Biological Material

This study used the bacterial strain *P. freudenreichii* T82, which was obtained from the pure culture collection of the Department of Food Biotechnology and Microbiology, Warsaw University of Life Sciences. The bacteria were stored in neutralized VL medium at 4 °C and frozen (in a glycerol environment, −80 °C). 

The pomace consisted of waste derived from various Polish varieties of apples that were pressed. It was stored at 20 °C, and its sugar content was determined by the enzymatic method using the Sucrose/D-Fructose/D-Glucose Assay Kit from Megazyme (according to the manufacturer’s instructions attached to the purchased test). The total nitrogen and protein content was determined by the Kjeldahl method. 

Potato wastewater was prepared from potatoes of the Irga variety. The preparation was carried out according to the methodology developed based on the technological process of obtaining potato starch from starch plants. Washed potatoes were mechanically comminuted, and cell juice was separated from the potato pulp. Starch residues from the wastewater were removed by centrifugation at 3000 rpm for 20 min (Roto Silenta 630 RS, Hettich Zentrifugen, Tuttlingen, Germany). The potato protein was precipitated by acidifying the wastewater with a hydrochloric acid solution to pH 5.0 and heating the resulting solution at 117 °C (10 min). Protein was separated from the solution by filtration, and the obtained potato wastewater was sterilized (121 °C for 20 min). Finally, the wastewater was stored in glass bottles at room temperature until use. The sugar and nitrogen content was determined as described above.

### 3.2. Culture Media

The bacteria were propagated in the VL medium (Btl, Warsaw, Poland) of the following composition: glucose (1 g/L), meat extract (3 g/L), peptone (10 g/L), sodium chloride (5 g/L), yeast extract (5 g/L), and L-cysteine hydrochloride (0.40 g/L). The inoculum medium was sterilized in an autoclave (20 min at 117 °C) before inoculation. The active acidity of the medium was set to 7.0 by adding 20% NaOH. 

The apple pomace and/or potato wastewater-based media were prepared as follows. One liter of distilled water or potato wastewater (depending on the medium variant) was added to 1 kg of pomace in glass bottles (1:1 m/v), and the whole solution was heated (extracted) for 30 min at 75 °C. After heating, insoluble materials were removed by filtration. This was followed by centrifugation (3500 rpm, 10 min, Roto Silenta 630 RS, Hettich Zentrifugen, Tuttlingen, Germany), and the supernatant (extract) was used to prepare the experimental media as shown in Table 3. The prepared media were sterilized at 121 °C for 15 min, and their pH was adjusted to 7.0 using 20% NaOH. 

### 3.3. Culture Conditions

Inoculation cultures were carried out in 250 mL Erlenmayer flasks, containing 100 mL of VL liquid medium, under static conditions at 30 °C. Bacterial multiplication was allowed until the suspension reached the optical density of 2.0, which was measured spectrophotometrically every 12 h. The obtained inoculum was used to inoculate liquid experimental media in a given series. Prior to inoculation of the production media, the inoculum cultures were centrifuged. For this purpose, 40 mL of the postculture fluid was collected in 50 mL tubes, and the samples were centrifuged (10,000 rpm, 10 min, Centrifuge 5804R, Eppendorf, Hamburg, Germany). The supernatant was decanted from the pellet, and the biomass was suspended in a suitable test medium. 

The experimental cultures were carried out under static conditions in 500 mL Erlenmayer flasks containing 250 mL of the liquid medium. The inoculum accounted for 10% of the volume of the production medium. Cultures were performed for 120 h at 37 °C. The media were neutralized with 20% NaOH at an interval of 24 h. Samples for analysis were taken at 0, 24, 48, 72, 96, and 120 h of the process. Cultures were carried out simultaneously in three flasks (flask 1—24 and 48 h, flask 2—72 and 96 h, flask 3—120 h) to minimize the impact of the depleting medium on the metabolic activity of the bacteria.

### 3.4. Analytical Methods

The cellular biomass yield was determined by weighing. The sugar content (converted into glucose) in the tested media and culture fluid was determined spectrophotometrically at λ = 550 nm (V-1200 Spectrophotometer, VWR, Leuven, Belgium) using 3,5-dinitrosalicylic acid (Miller method). For determining the content of sucrose, the samples were hydrolyzed. 

Trehalose determination in cellular biomass was performed using the Megazyme Trehalose Assay Kit enzyme assay (Wicklow, Ireland). Briefly, 100 mg of frozen cellular biomass were weighed into Eppendorf tubes of known weight. B-PER (Thermo Scientific, Waltham, Massachusetts, MA, USA) reagent (0.5 mL) was added to each sample to disrupt cell structures and extract trehalose. The samples were incubated for 30 min at room temperature (mixed every 5 min to intensify the process) and centrifuged at 10,000 rpm for 10 min (Centrifuge 5804R, Eppendorf, Hamburg, Germany). The obtained supernatant was used to determine the trehalose content. Then, the sediment was dried (80 °C, 24 h), and the dried biomass was used to calculate the mass of trehalose. The rest of the procedure was carried out according to the instructions provided with the purchased test (Megazyme Trehalose Assay Kit enzyme assay). 

The analysis of the produced acids—propionic and acetic acids—was carried out using a gas chromatograph equipped with a flame ionization detector (Trace 1300 Gas Chromatography, Thermo Scientific, Waltham, MA, USA). To release free organic acids from the salts of propionic and acetic acids (resulting from regular alkalization of the media during fermentation), 25% sulfuric acid (VI) was added to postculture fluids (the volume depended on the amount of 20% NaOH introduced into the medium during the culture). The carboxylic acid fraction was extracted from the media using a mixture of hexane and diethyl ether (1/1, *v*/*v*). Chromatographic separation was performed on the ZB-WAXplus column (30 m × 0.25 mm × 0.25 µm). The qualitative analysis of acids was performed by comparing the retention times of the tested samples with those of the standards. Quantitative calculations were made using the analytical standard (undecanoic acid). From the obtained results, acid production efficiency, productivity, and the weight ratio of propionic acid to acetic acid were determined.

### 3.5. Statistical Analysis

The results obtained in three independent series were statistically analyzed in the Statistica program (version 10.0, StatSoft, Poland). The normal distribution of the data was determined by the Shapiro–Wilk test, and the homogeneity of variance by the Levene test. The significance of differences between the mean values was analyzed by one-way analysis of variance and Tukey’s test. The analyses were performed at the significance level of α = 0.05.

## 4. Conclusions

The results of this study showed that the media consisting of apple pomace extract and potato wastewater constituted a favorable environment for the cultivation of PAB. This was evidenced by the relatively high efficiency of propionic acid production (compared to other scientific reports) and high biomass yield of the *P. freudenreichii* T82 strain. Lower trehalose accumulation in the bacterial cell biomass, compared to the literature data, proves that the cultivation conditions applied in the study did not have any extreme stressful effect on the tested strain. Thus, the study shows that PAB can be successfully grown in media containing several waste materials. It also confirmed that potato wastewater can serve as a rich source of nitrogen and thus a replacement for expensive supplements such as peptone or yeast extract. This may allow effective waste management, improving the quality of the natural environment, and lowering the price of biotechnologically produced propionic acid.

## Figures and Tables

**Table 1:** Characteristics of waste materials.

Component/Parameter	Apple Pomace Extract	Potato Wastewater
Total sugars (g/100 mL)	4.54 ± 0.11	0.51 ± 0.08
Glucose (g/100 mL)	1.91 ± 0.11 (42%)	0.24 ± 0.02 (47%)
Fructose (g/100 mL)	2.42 ± 0.14 (53%)	0.27 ± 0.00 (53%)
Saccharose (g/100 mL)	0.21 ± 0.08 (5%)	0.00 ± 0.00 (0%)
Total protein (g/100 mL)	0.138 ± 0.020	1.338 ± 0.057
Nitrogen (g/100 mL)	0.022 ± 0.030	0.214 ± 0.009
pH	3.82	5.09
C/N	39.2:1	0.5:1

**Table 2:** Parameters of propionic acid fermentation.

Medium	PA Yield	AA Yield	PA Productivity	AA Productivity	P/A Ratio
	(g/g)		(g/Lh)		
I	0.18	0.08	0.011	0.005	2.37:1
II	0.41	0.12	0.062	0.019	3.53:1
III	0.39	0.18	0.053	0.025	2.15:1
IV	0.21	0.10	0.019	0.009	2.05:1
V	0.44	0.11	0.121	0.030	3.99:1
VI	0.42	0.16	0.106	0.042	2.53:1

**Table 3:** Composition of media.

Compound	Medium					
	I *	II *	III *	IV	V	VI
Apple pomace (kg/L)	1.00	1.00	1.00	1.00	1.00	1.00
Potato wastewater (L)	-	-	1.00	-	-	1.00
Yeast extract (g/L)	-	10.00	-	-	20.00	-
Peptone (g/L)	-	5.00	-	-	10.00	-
Potassium dihydrogen phosphate (g/L)	-	2.50	-	-	2.50	-
Dipotassium hydrogen phosphate (g/L)	-	1.50	-	-	1.50	-
L-cysteine hydrochloride (g/L)	0.40	0.40	0.40	0.40	0.40	0.40
Biotin (mg/L)	-	0.20	-	-	0.20	-
Distilled water (L)	1.00	1.00	-	1.00	1.00	-
C/N **	36.6:1	4.6:1	3.6:1	34.9:1	4.4:1	3.5:1

* Extracts were diluted 2x to obtain media with a lower concentration of carbon sources. ** The calculations took into account glucose, fructose (reducing sugars), sucrose, and nitrogen/the study did not discuss the consumption of sucrose by the tested strain, because *P. freudenreichii* T82 does not assimilate and does not ferment this disaccharide.

## Data Availability

Data available on request.

## Sample Availability

Samples are available from the authors.
